# Maternal complications and associated factors among mothers who underwent a cesarean section at Gebretsadik Shewa general hospital: an institution based cross-sectional study

**DOI:** 10.3389/fgwh.2023.1091863

**Published:** 2023-08-09

**Authors:** Ketemaw Negese, Dereje Zeleke Belachew

**Affiliations:** Department of Midwifery, College of Medicine and Health Sciences, Mizan–Tepi University, Mizan Aman, Ethiopia

**Keywords:** magnitude, cesarean section, associated factors, maternal complication, Gebretsadik Shewa general hospital

## Abstract

**Introduction:**

Cesarean sections have played a major role in lowering maternal morbidity and mortality rates, but are a major concern in developing countries. This study aims to assess the magnitude of maternal complication and its associated factors among women who underwent a cesarean section at Gebretsadik Shewa general hospital, Southwest Ethiopia.

**Method:**

A hospital-based cross-sectional study was conducted in Gebretsadik Shewa general hospital. Data were extracted from 382 mothers' medical charts, retrieved from the labor and operations theatre log book registry using systematic random sampling technique. The extracted data was coded and entered into Epi Data version 3.0, and exported to Statistical Package for Social Sciences (SPSS) version 20 for analysis. Logistic regression analysis was conducted and significance and strength association was determined considering AOR with a 95% confidence level.

**Result:**

A total of 382 mothers' charts were reviewed; however, 368 charts were eligible for data entry. The age of the participants ranges between 16 and 42 years with mean and standard deviation of 26.1 ± 4.8 years. Maternal complication rate was 30.4% [95% CI: 25.8- 35.1]. Surgical site infection (10.3%), anemia (6.5%) and intraoperative bleeding (4.6%) were the most common. Multivariable logistic regression analysis showed that no antenatal care follow up, medical illness during pregnancy, emergency cesarean section and not receiving a prophylactic antibiotic were statistically associated with maternal complications.

**Conclusion:**

The incidence of maternal complication following cesarean delivery was unduly high. Community based education about antenatal care follow up and its importance should be further strengthened for favorable maternal and fetal outcomes.

## Introduction

Cesarean section (CS), also known as cesarean delivery, refers to the delivery of the fetus, placenta and membrane through abdominal and uterine incision after fetal viability (1). Globally, the cesarean section rate is unevenly distributed and results in 21.1% of abdominal deliveries. Africa accounts for 5.0% cesarean deliveries in the world (2).

Maternal complications related to cesarean delivery are defined as the presence of either one of intraoperative or postoperative surgical complications. The American College of Obstetricians and Gynecologists (ACOG) reported that cesarean delivery significantly increased a woman's risk of pregnancy-related fatality (35.9 deaths per 100,000 live births) compared to a woman who delivered vaginally (9.2 deaths per 100,000 live births) (3). A study conducted in Australia reported that among 43 maternal deaths, 31 were related to cesarean deliveries (4).

A prospective study conducted in Finland examined the complication rate among 2,500 women who underwent a cesarean section within a 6 months time frame. The rate of serious complications (serious complications were defined as follows: more than 1,500 ml blood loss, need for blood transfusion, hysterectomy, need for another surgery, septicemia, blood clots, pulmonary edema, and pneumonia) for all cesarean sections was 10.4% (5). When groups are stratified, emergency and crash-emergency cesarean sections had far higher serious complication rates; 11.7% and 25% respectively (5).

In Africa, a cesarean section is performed under harmful conditions in order to save the mother and her fetus (6). However, the maternal mortality rate related to caesarean section is nearly 5 times higher than that of vaginal delivery (7). There is disparity regarding the prevalence of cesarean sections and maternal morbidity and mortality (8). Studies conducted in Nigeria found that despite an increase in cesarean sections, the maternal mortality rate is reduced only from 831.9 to 708 per 100,000 live births (9, 10) and 190 per 100,000 live births in South Africa (11). About 50% of mothers develop complications such as post-operative wound infection, anemia requiring blood transfusion, respiratory tract infection, puerperal psychiatric disorders, septicemia, and wound dehiscence within the first 7 days of operation (10).

The increased risk of maternal morbidity and mortality associated with increased rates of cesarean sections underlie the growing concerns of health care providers. Obstetricians in the United Kingdom (UK) have conducted studies to address this issue. A woman delivered via cesarean section has developed a uterine scar. This scar has important implications for future pregnancies as the patient is predisposed to uterine rupture, placenta previa and placenta accreta (12).

A facility based survey of 797 health facilities in Ethiopia indicated that the cesarean delivery rate was 1.9% with regional rates varying from 0.2% to 9% (13, 14). The overall institutional rate was 18%, which varied between 46% in the private sector and 15% in the public sector. Majorities were performed for emergency cesarean section and maternal indications. More than 50% of the cases were operated on within a 30 min to 5 h interval between decision and delivery. Prophylactic antibiotics were administered to 94% of reviewed cases; however, 12% of the cases reported post-operative wound infection, and there were two maternal deaths (14).

A retrospective study conducted in Tigray, Northern Ethiopia, also reported higher adverse maternal outcomes in 11 hospitals of the region. Of the total 2,835 cesarean deliveries, there were 17 (0.6%) maternal and 506 (17.8%) fetal deaths (15). The cesarean section rate in Jimma Hospital was 28.1%. The most common indications were cephalopelvic disproportion (14.1%) and previous cesarean section (3.8%). Fever (6.21%) and fistula (2%) were complications following cesarean section (16).

Ethiopia implements various strategies to overcome these adverse maternal outcomes including strengthening health facilities, providing training for health care providers, including health extension workers (HEW), so that at least each low and mid-level facility can provide basic emergency obstetrics and neonatal care (BEMONC) to early identify indications and reduce the cesarean section rate (17, 18). Gebretsadik Shewa general hospital is a health facility equipped to provide comprehensive emergency obstetric care including cesarean sections and blood transfusion services.^1^

Various studies in Ethiopia indicated that maternal complication following cesarean section is higher and mainly related to medical illnesses during pregnancy, urgency of indication, and operation related factors (type of cesarean section performed, length of cesarean section and the type of anesthesia used) (19, 20). It is also found that rural residency of the mother, prolonged labor, gestational age, previous history of obstetric complication, parity, and no antenatal care follow up are the contributing factors for an increased maternal complication following cesarean delivery (20–23).

To the best of our knowledge, however, the magnitude of maternal complication and its associated factors in women who underwent a cesarean section at Gebretsadik Shewa general hospital has not yet been studied. This study was aimed to assess the magnitude of and factors associated with maternal complications following cesarean sections at Gebretsadik Shewa general hospital.

## Methodology

### Study area and period

This study was conducted at Gebretsadik Shewa general hospital from November 1, 2021 to January 1, 2022. It is located in Bonga town, a municipality for Kaffa zone, Southwest Ethiopia Region. It is located 445 kms south of Addis Ababa, the capital city of Ethiopia. It provides health care services for a catchment population of 4.5 million mainly from Kaffa zone and surrounding areas. It provides service for patients referred from health centers and district hospitals. This hospital has 545 workers (328 health professionals and 217 supporting staff). It serves approximately 70,517 patients of all types annually. Gebretsadik Shewa general hospital has six departments and 115 beds. Obstetrics and Gynecology is one of the major departments in the hospital. The department has three specialist physicians in Obstetrics and Gynecology, 5 general practitioners, 2 Integrated Emergency Obstetric care Surgeons (IEOSs) and 20 midwives. It provides maternal health care services free of charge. It has a total of 40 beds and 4 delivery coaches. Cesarean section is the most common surgical procedure performed by the Obstetrics and Gynecology department. The average quarterly total number of cesarean deliveries was 199 per 715 of the total deliveries in 2021/22. However, the department does not have its own separate operating theater room.

### Study design

A hospital-based cross-sectional audit of patient records was conducted to estimate the magnitude of maternal complication and identify associated factors among mothers who underwent a cesarean section/delivery from September 1, 2018 to August 30, 2021.

## Source and study population

### Source population

All mothers who gave birth via cesarean section in Gebretsadik Shewa general hospital.

### Study population

All records of mothers who gave birth via cesarean section in Gebretsadik Shewa general hospital from September 1, 2018 to August 30, 2021.

## Inclusion and exclusion criteria

### Inclusion criteria

All cesarean deliveries performed after the period of fetal viability (> = 28 weeks) were included for this study.

### Exclusion criteria

Cesarean deliveries with complications referred from other health facility, lost charts and lacking complete documentation were excluded from the study.

## Sample size determination

Sample size was computed using a single population proportion formula by considering the following assumptions; *p* = 38% (19), *z* = 1.96, *CI* = 95% and *d* = 5%; Where

*p* = Population proportion.

*z* = Standard normal distribution for 95% confidence level.

*d* = Margin of error and.

*CI* = Confidence interval.

The final sample size for the study was computed after using adjustment formula and 10% non-response rate, with final sample size (nf) = 382.

## Sampling procedure

The study population (all records of cesarean deliveries from September 1, 2018 to August 30, 2021) was retrieved from the labor and operation theatre log book registry. They were listed in order based on the date and year of the operation performed (starting from September 1, 2018) to form the sampling frame of the study. There were 2,413 total cesarean deliveries in the hospital from September 1, 2018 to August 30, 2021. Sampling/skip interval, *k* was then calculated by dividing the total number of eligible mothers who delivered via cesarean section (2,413) by the final sample size (382), providing sampling/skip interval, *k* = 6. The first chart/patient record was randomly selected using lottery method and subsequent records were identified using systematic random sampling in every sixth case after arranging the study units in order of the date of operation. The chart/record of the patient whose cesarean delivery procedure was performed next to the case selected for data extraction was considered if the selected patient's chart/record was missed or lost.

## Variables

### Dependent variable

Maternal complication following cesarean section/delivery.

### Independent variables

Socio demographic factors (age, residency, religion, occupation).

Obstetrics related factors (antenatal care, parity, previous CS scars, urgency of surgery, medical illness during pregnancy, duration of labor, and gestational age at delivery).

Operation related factors (type of cesarean section performed, type of anesthesia used, indications for cesarean section, prophylactic antibiotic and duration of surgery).

## Operational definition

Maternal complications following cesarean delivery include the presence of one or more of the following signs and symptoms on a patient during the operation or following cesarean delivery within 7 days of surgery: infection, fever, wound dehiscence, hemorrhage (intra partum or postpartum), organ injuries, anemia, respiratory tract infection, and postpartum cardiomyopathy.

## Data collection tools and procedures

Data was collected from client records using a designed, structured data extraction format containing important preoperative, intraoperative and postoperative data. It was prepared in English and developed after review of various literatures and books and through observing clinical and operating records, delivery room charts, and institutional annual reports. Data was extracted by four midwives and direct supervision was made by two Integrated Emergency Obstetric care Surgeons (IEOS) working in Obstetrics and Gynecology department.

## Quality assurance technique

One day of training was given for data collectors and supervisors regarding the data extraction procedure. Before proceeding to data extraction, the format was pretested to check for its consistency and the ability of data collector's performance. It was conducted on 5% (18) samples of cesarean section cases in Mizan–Tepi University Teaching Hospital (MTUTH) and necessary modification was made based on pretest result. The final data extraction format was then checked by data collectors and supervisors on daily basis for its completeness, consistency, accuracy and validity of the data. Both supervisors and principal investigators provided day-to-day onsite supervision in the whole data extraction period of the study.

## Data analysis and processing

The extracted data was coded in a pre-arranged data sheet. It was entered into Epi Data version 3.0 and exported to Statistical Package for Social Sciences (SPSS) version 20 for analysis. Bivariable analysis was performed to determine the association between different factors with the outcome variable. Variables which were significant on bivariable analysis (*p*-value ≤ 0.25) were entered for multivariable logistic regression analysis. Variables with *p*-value ≤ 0.05 using multivariable logistic regression analysis were considered statistically significant associated factors with the outcome variable. Odds Ratio (OR) with 95% confidence interval (CI) was used to determine the association between independent and dependent variables. Model of fitness was checked with Hosmer and Lemeshow goodness of fit test setting *p*-value = 0.895. Multicollinearity test was performed and all variables had variance inflation factor (VIF} less than 10. Finally, descriptive statistics were computed and the results were presented in the form of texts, tables and figures.

## Ethics approval and consent to participate

Ethical approval for this study was obtained from the Research Ethics Committee of Mizan–Tepi University, College of Medicine and Health Sciences; with a reference number MTU/REC/00975/DA/2021. Written and signed consent was obtained from the administrative staff of Gebretsadik Shewa general hospital after a permission letter was written from Medicine and Health Sciences College, Mizan–Tepi University.

## Result

### Socio-demographic characteristics of study subjects

A total of 368 cases were involved for this study, providing 96.3% response rate. This was due to incomplete data for fourteen cases which were purposely excluded from analysis. The age of study participants ranges between 16 and 42 years, with a mean and standard deviation of 26.1 ± 4.8 years. The dominant age group was in the range between 25 and 29 years and the majority (67.1%) were from a rural residence. About 97.8% of mothers were married (Table 1).

**Table 1:** Socio-demographic characteristics of mothers who underwent a caesarean section at Gebresadik Shewa general hospital (*n* = 368).

Variables	Category	Number	Percent (%)
Age in years	<20	34	9.2
	20–24	127	34.5
	25–29	138	37.5
	30–45	69	18.8
Place of residency	Urban	121	32.9
	Rural	247	67.1
Marital status	Married	360	97.8
	Others	8	2.3

### Obstetrics related factors

Mothers who were referred from other health institutions numbered 294 (79.9%) and the remaining 74 (20.1%) were self-referred. Regarding parity, 170 (46.2%) were primipara, 151 (41%) were multipara, and 47 (12.8%) were grand multipara. About 84.8% were booked for antenatal care and 87.2% were term (≥37 weeks) and 6% preterm (<37 weeks) (Table 2).

**Table 2:** Obstetric factors of mothers who underwent a caesarean section at Gebretsadik Shewa general hospital (*n* = 368).

Variables	Category	Frequency	Percent (%)
Parity	Primipara	170	46.2
	Multipara	151	41
	Grand-multipara	47	12.8
ANC follow up	Yes	312	84.8
	No	56	15.2
Referral status	Yes	294	79.9
	No	74	20.1
Gestational age	Preterm	22	6
	Term	321	87.2
	Post term	25	6.8
Medical illness	No	309	84
	Yes	59	16
Number of children	Single	344	93.5
	Multiple	24	6.5

**Figure 1:**
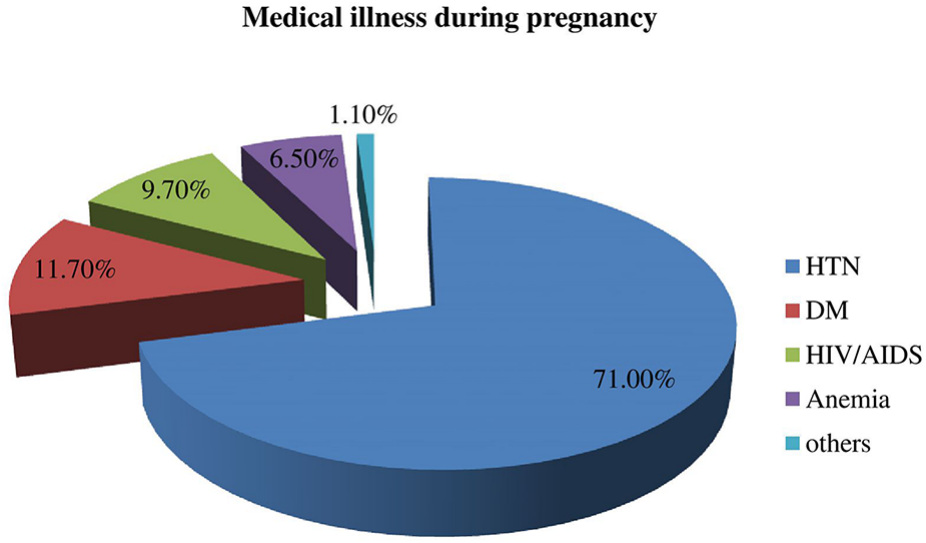
Medical illnesses during pregnancy among mothers who underwent a caesarean section in Gebretsadik Shewa general hospital.

Fifty-nine mothers had developed medical illnesses during their pregnancy period. The number of mothers with hypertension was 42 (71%), followed by diabetes 7 (11.7%), HIV/AIDS 6 (9.7%), and anemia 4 (6.5%) respectively (Figure 1).

### Operation related factors

The prevalence of cesarean section at Gebretsadik Shewa general hospital was 20.9%. The majority 327 (88.9%) underwent emergency cesarean section while the rest were elective (11.1%). Regarding the type of anesthesia, 298 (81%) were performed with spinal anesthesia and the rest 70 (19%) with general anesthesia. Mothers who underwent surgery for more than 60 min were 25.3% and mothers who did take antibiotics before surgery were 207 (56.3%). Regarding the types of surgery performed, 346 (94%) were delivered with lower uterine segment transverse caesarean section (LUSTCS), followed by classical 17 (4.6%), and inverted T 5 (1.4%) respectively (Table 3).

**Table 3:** Operation related factors for mothers who underwent a caesarean section at Gebretsadik Shewa general hospital (*n* = 368).

Variables	Category	Frequency	Percent (%)
Circumstance of surgery	Elective	41	11.1
	Emergency	327	88.9
Frequency of surgery	Primary	321	87.2
	Repeat	47	12.8
Duration of surgery (minutes)	<30	12	3.3
	30–60	263	71.4
	>60	93	25.3
Antibiotics before surgery (CS)	Yes	207	56.3
	No	161	43.7
Decision to delivery	<1 h	60	17.6
	1–2 h	191	56
	≥ 2 h	90	26.4
Types of caesarean section	LUSTCS	346	94
	Classical	17	4.6
	Inverted T	5	1.4
Type of anesthesia	Spinal anesthesia	298	81
	General anesthesia	70	19

### Indications for surgery (cesarean section)

The leading indication of caesarean section at Gebretsadik Shewa general hospital was non-reassuring fetal heart rate pattern (NRFHRP) (31.3%), followed by cephalopelvic disproportion (CPD) (28.5%), obstructed labor (OL) (12.2%), previous cesarean section (9.5%), antepartum hemorrhage (APH) (8.2%), hypertensive disorders of pregnancy (HDP) (4.6%), and others (transverse lie, cord presentation, previous myomectomy, and tumor previa) were 5.7% (Table 4).

**Table 4:** Indications for caesarean section at Gebretsadik Shewa general hospital (*n* = 368).

Indications for caesarean section	Frequency	Percent (%)
CPD	105	28.5
NRFHRP	115	31.3
Hypertensive disorders	17	4.6
Obstructed labor	45	12.2
Previous cesarean section	35	9.5
APH	30	8.2
Others	21	5.7

## Maternal outcomes

**Figure 2:**
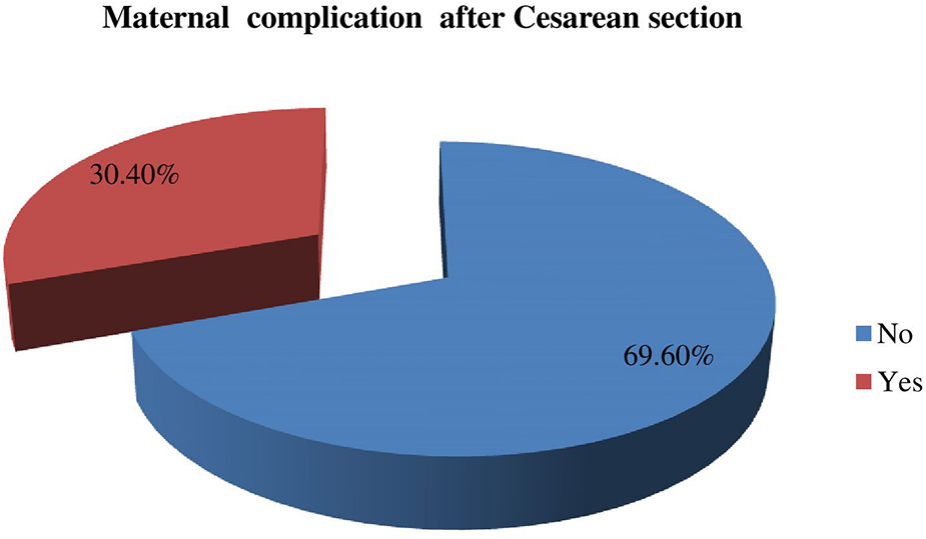
Magnitude of maternal complication among mothers who underwent a caesarean section in Gebretsadik Shewa general hospital.

The number of study subjects who developed maternal complications both in the intraoperative (during surgery) and following cesarean section was 112 (30.4%); [95% CI: 25.8–35.1] (Figure 2).

### Intraoperative complication

**Figure 3:**
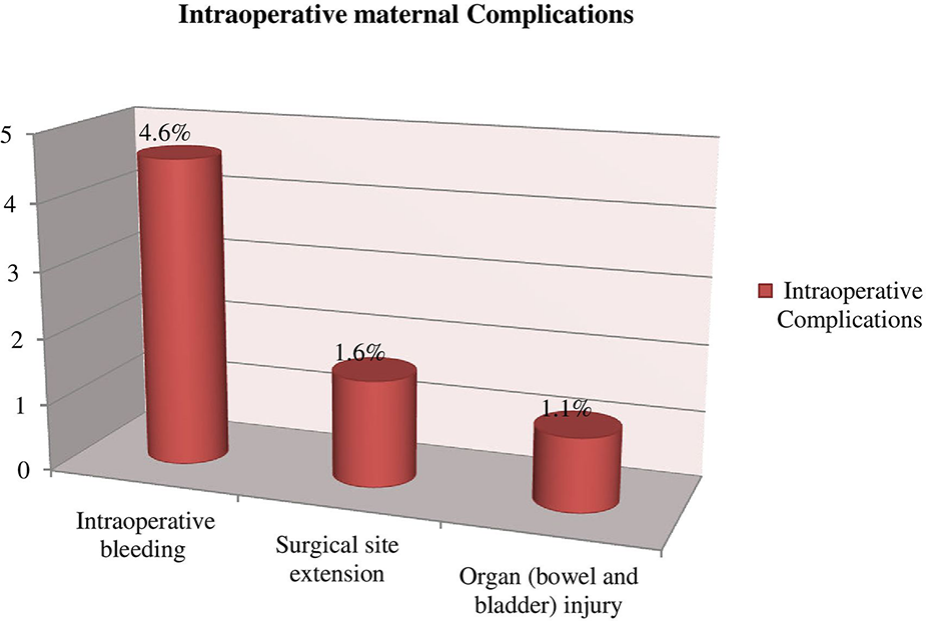
Intraoperative maternal complications among mothers who underwent a caesarean section in Gebretsadik Shewa general hospital.

Of those mothers who developed maternal complications, 28 (7.3%) occurred in the intraoperative period (time of surgery). These include intraoperative bleeding which accounts for 4.6% followed by surgical site extension (1.6%) and organ (bowel and bladder) injury (1.1%) respectively (Figure 3).

### Postoperative (post-surgery) complication

**Figure 4:**
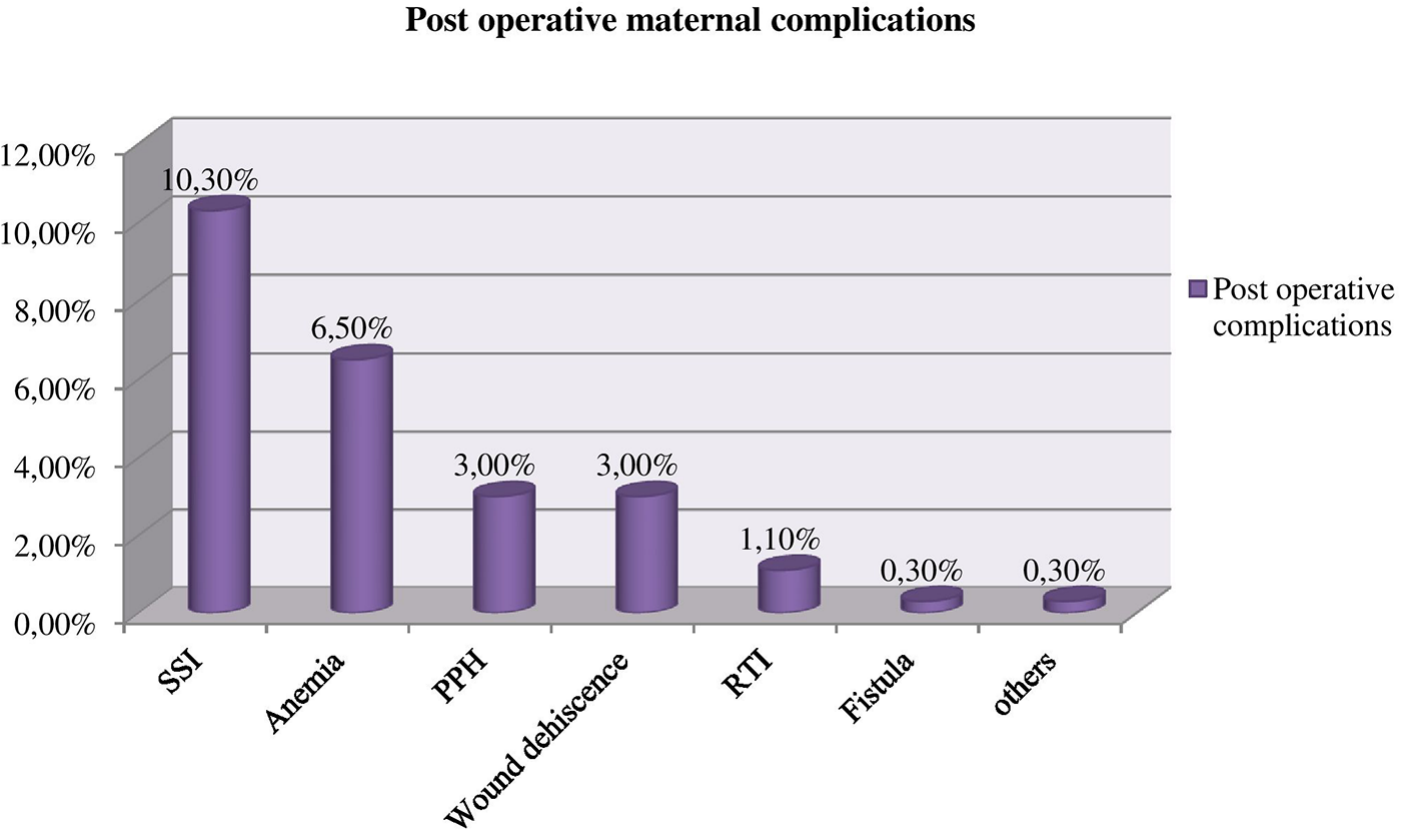
Postoperative maternal complications among mothers who underwent a caesarean section in Gebretsadik Shewa general hospital.

Out of the total study subjects, 90 (24.5%) maternal complications developed after surgery/cesarean delivery was completed. Surgical site infection (SSI) was the most common complication (10.3%), followed by anemia (6.5%), postpartum hemorrhage (PPH) (3%), wound dehiscence (3.0%), respiratory tract infection (RTI) (1.10%), fistula (0.3%) and others (0.3%) respectively. There were two postoperative maternal deaths due to multiple organ failure secondary to septic shock and cardiogenic shock secondary to postpartum cardiomyopathy (Figure 4).

## Factors associated with maternal complication at Gebretsadik Shewa general hospital

Variables such as residence, referral status, duration of labor, type of anesthesia used for surgery, history of antenatal care follow up, medical illness during pregnancy, urgency for surgery, and prophylactic antibiotic administration were significantly associated with adverse maternal outcome in bivariable analysis (*p*-value ≤ 0.25). However, variables such as history of antenatal care follow up, medical illness during pregnancy, urgency for cesarean delivery and prophylactic antibiotic administration remained independent factors contributing towards maternal complication using multivariable logistic regression (*p*-value ≤ 0.05).

Accordingly, mothers who did not have a history of antenatal care follow up and mothers with medical illness during pregnancy were 2.95 [AOR: 2.95; 95% CI: 1.19–7.29] and 4.28 [AOR: 4.28; 95% CI: 1.58–11.61] times more likely to develop maternal complications compared with mothers with a history of antenatal care follow up and without medical illness history respectively.

Similarly, pregnant women who underwent emergency cesarean section and who did not take prophylactic antibiotics before surgery were 7.09 [AOR: 7.09: 95% CI: 1.19–45.5] and 3.20 [AOR: 3.20; 95% CI: 1.43–6.94] times more likely to develop adverse maternal outcomes than those who underwent elective cesarean section and took prophylactic antibiotics before surgery respectively (Table 5).

**Table 5:** Factors associated with maternal complication following caesarean delivery at Gebretsadik Shewa general hospital (*n* = 368).

Predictor variables	Maternal complication		COR (95% CI)	AOR (95% CI)
	Yes	No		
Address				
Rural	84	163	1.54 (1.04–2.81)*	1.46 (0.6–3.41)
Urban	28	84	1	1
ANC follow up				
No	23	33	1.74 (0.97–3.13)*	2.95 (1.19–7.29)**
Yes	89	223	1	1
Referral status				
No	58	16	16 (8.6–30.1)*	2.53 (0.92–6.91)
Yes	54	240	1	1
Medical illness				
Yes	35	24	4.41 (2.46–7.84)*	4.28 (1.58–11.61)**
No	77	232	1	1
Duration of labor				
No labor	17	42	0.19 (0.09–0.41)*	0.21 (0.92–2.35)
≥ 24 h	51	25	0.11 (0.064–0.204)*	3.19 (0.67–15.2)
< 24 h	44	189	1	1
Urgency of surgery				
Emergency	107	220	3.5 (1.33–9.17)*	7.09 (1.19–45.5)**
Elective	5	36	1	1
Type of anesthesia				
Spinal	73	225	1	1
General	39	31	0.26 (0.15–0.44)*	0.5 (0.2–1.26)
Antibiotics				
No	77	84	4.5 (2.79–7.26)*	3.2 (1.43–6.94)**
Yes	35	172	1	1

COR, crude odds ratio; AOR, adjusted odds ratio.

* *p* < 0.25 in bivariate.

** *p* < 0.05 in multivariate.

## Discussion

The magnitude of maternal complication in this study was 30.4% [95% CI: 25.8–35.1]. This is higher compared with studies conducted in Tigray (19.3%) (24), Oromia (20.5%) (25) and Debre Birhan (16.5%) (26). The possible reason might be due to the difference in level of hospitals. In this study, more cesarean deliveries were performed on mothers with history of previous cesarean section and even in a single episode of fetal distress due to the absence of specialists who can manage complicated labor using other modes of delivery. It is also greater than the finding in Germany (10.5%) (27). This might be due to a more pronounced and advanced obstetrics emergency care in Germany. Maternal complications as a result of cesarean sections are unevenly distributed around the globe and are lower for developed countries (6).

However, this finding was relatively lower than findings in Arba-minch (38.2%) (19), Bahirdar (44.04%) (28) and Hawassa (56%) (29). The possible explanation might be due to the discrepancy in number of obstetrics emergency cases for hospitals. Bahirdar and Hawassa hospitals are fourth tier health institutions and may include cases referred with complications from district and general hospitals. Finding inconsistency with Arba-minch general hospital can be also due to the difference in study design. The study in Arba-minch hospital was conducted with retrospective cohort study design.

In this study, intraoperative complications such as intra operative bleeding (4.6%), surgical site extension (1.6%) and organ (bowel and bladder) injury (1.1%) were the most common complications that occurred during surgery, while surgical site infection (10.3%), anemia (6.5%), postpartum hemorrhage (3.0%), wound dehiscence (3.0%) and respiratory tract infection (1.1%) were most common after the cesarean section was complete. Bladder and bowel injuries were lower compared with the finding in Arba-minch hospital (5.5%) (19). Studies conducted in Finland did not find organ injury (5). This can be due to the difference in antenatal care follow up of participants. More participants from Arba-minch hospital had no history of antenatal care follow up and may be more likely to have an emergency cesarean delivery which in turn increases the risk of organ injury. The other possible explanation might be due to lacking a specialist who is skillful in managing complicated labor using modes of delivery other than cesarean section.

Antenatal care follow up is found to be a statistically significant associated factor with maternal complication in this study. Mothers who did not have a history of antenatal care follow up were 2.95 times more likely to develop maternal complications than their counterparts. This is consistent with the finding in Attat hospital (30). This could be due to the fact that mothers who do not attend antenatal care are not screened and identified early for high risk pregnancy and therefore timely intervention.

A medical disorder during pregnancy was also another statistically significant associated factor with maternal complication in this study. This is in agreement with studies in Arba-minch and Gelemso hospitals (24, 31). Pregnant mothers with medical illness were 4.28 times more likely to develop maternal complications. The possible reason might be because pregnancy is a period of immune suppression, providing a weaker immune response to disease, exposing the mother to an increased likelihood of post operative wound infection.

Studies conducted at Bertha Gxawa Hospital and hospitals in Finland found that emergency cesarean section was a significant factor which contributes for adverse maternal outcome (32, 33). A study which was conducted to assess fetal and neonatal outcome in emergency vs. elective cesarean section in Nepal concluded that post-operative wound infection, post-partum hemorrhage and need for blood transfusion were higher for cesarean section than elective cesarean groups (34). The finding of this study also supported findings of these hospitals. Mothers who underwent an emergency cesarean section were 7.09 times more likely to develop maternal complications than mothers who had a planned/elective cesarean section. The reason might be due to insufficient pre-arranged operation theater room optimization for patients requiring emergency surgery.

Mothers who did not take prophylactic antibiotics before surgery were 3.20 times more likely to develop maternal complications in this study. It was supported by findings in Gelemso and Hawassa hospitals (31, 35). These studies documented that administration of first dose antimicrobial prophylaxis within 1 h before surgery reduces the incidence of infection that develops as result of contaminations during surgery. This might be due to the fact that either low or poor potent serum antimicrobial levels that can fight against foreign microbials acquired during surgery for mothers who did not take prophylactic antibiotic.

Duration of labor greater than 24 h and general anesthesia were significantly associated at Gelemso and Gondar University Teaching Hospital (31, 36). However, there is no statistically significant association in this study. This might be due to the small sample size and difference in study design and study period.

## Limitations of the study

Maternal complication related factors such as Body Mass Index (BMI) and estimated blood loss were not registered in patients' medical charts and if fulfilled may affect the outcome. The result of this study was limited to only Gebretsadik Shewa general hospital and it is not generalized to other hospitals located in the region.

## Conclusion and recommendation

The magnitude of maternal complication following cesarean section was higher than non-cesarean deliveries. The study identified that mothers who did not have antenatal care follow up, had medical illnesses during pregnancy, delivered via emergency cesarean section and did not receive prophylactic antibiotics were factors statistically associated with maternal complication. Community based education about antenatal care follow up and its importance should be further strengthened for favorable maternal and fetal outcomes. Waiting rooms should be prepared at hospitals for pregnant mothers previously screened and identified as having a high risk pregnancy and mothers living in areas where access to health care service is difficult/poor. Operating theater rooms should be well organized, optimized and reserved for mothers seeking emergency cesarean section. Prophylactic antimicrobials/antibiotics must be administered before surgery and continue after surgery based on physician recommendation.

Finally, for researchers who are willing to conduct similar research, it is better to include Body Mass Index and the amount of total blood lost during and after surgery, which are not included in this study but may have a significant impact on the outcome.

## Data Availability

The raw data supporting the conclusions of this article will be made available by the authors, without undue reservation.

## Footnotes

^1^
Kaffa zone Health Department Communication Office report. Bonga town, Ethiopia. (2021)
